# Supplementary material for: Atypical E2Fs either Counteract or Cooperate with RB during Tumorigenesis Depending on Tissue Context
Source: Cancers (Basel). 2021 Apr 23;13(9):2033. doi: 10.3390/cancers13092033 (PMC8122802; doi:10.3390/cancers13092033)
Supplement: Supplementary file 1 [file cancers-13-02033-s001.zip › cancers-1161383-supplementary.pdf]

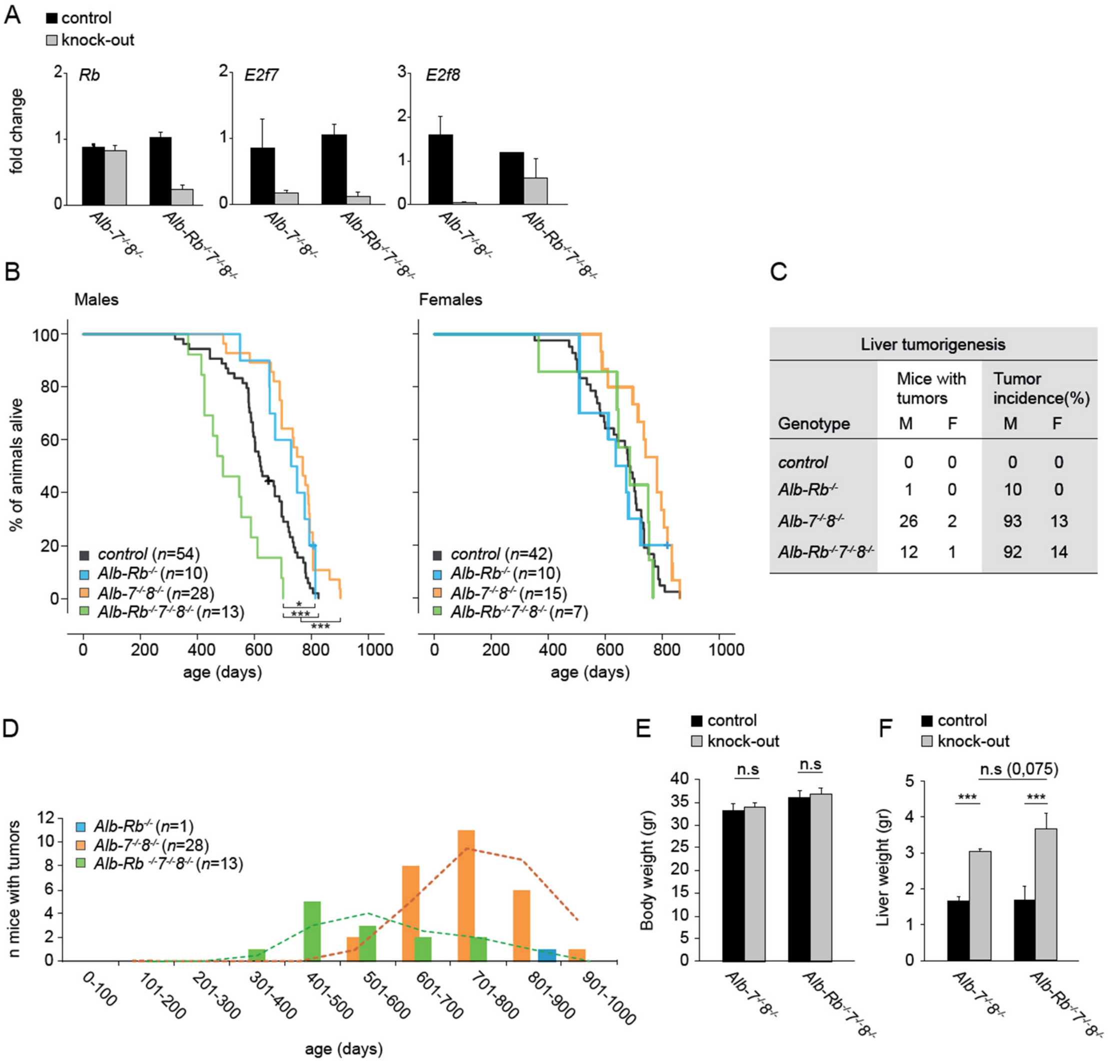

Figure S1: Atypical E2Fs and RB cooperate to prevent liver cancer. (**A**)Transcript levels of Rb, E2f7 and E2f8 from *Alb- 7-/-8-/-* and *Alb-Rb-/-7-/-8-/-* mice in non-tumor areas. Fold changes were adjusted to average of controls and Actb, Gapdh and Rsp18 were used to normalize the expression. Data represent average ± SEM (*n*= 4-5 mice). (**B**) Kaplan-Meier overall survival curves of males and females from *control*, *Alb-Rb-/-*, *Alb-7-/-8-/-* and *Alb-Rb-/-7-/-8-/-* mice. (**C**) Table indicating tumor incidence (%) of males and females of the indicated genotypes. Tumors collected at the end of life span. (**D**) Tumor latency distribution. Bar chars represent number of animals with tumors at the indicated age period. Dash lines indicate distribution of the data. (**E**) Body weights of *control* and *Alb-7-/-8-/-* and *Alb-Rb-/-7-/-8-/-* knock-out mice. Data represents average ± SEM (*n*= 15-20 *controls*; *n*=20 *Alb-Rb-/-7-/-8-/- ko* ; *n*=10 *Alb-7-/-8-/- ko*). (**F**) Liver weights of *control* and *Alb-7-/-8-/-* and *Alb-Rb-/-7-/-8-/-* ko mice. Data represents average ± SEM (*n*= 15-20 *controls*; *n*=20 *Alb-Rb-/-7-/-8-/- ko* ; *n*=10 *Alb-7-/-8-/- ko*).

Data information: in (B:survival and C: latency) Log Rang (Mantel-Cox) test and in (E and F) Mann-Whitney Rank Sum test ; n.s not significant, *p<0.05,**p<0.01, ***p<0.001

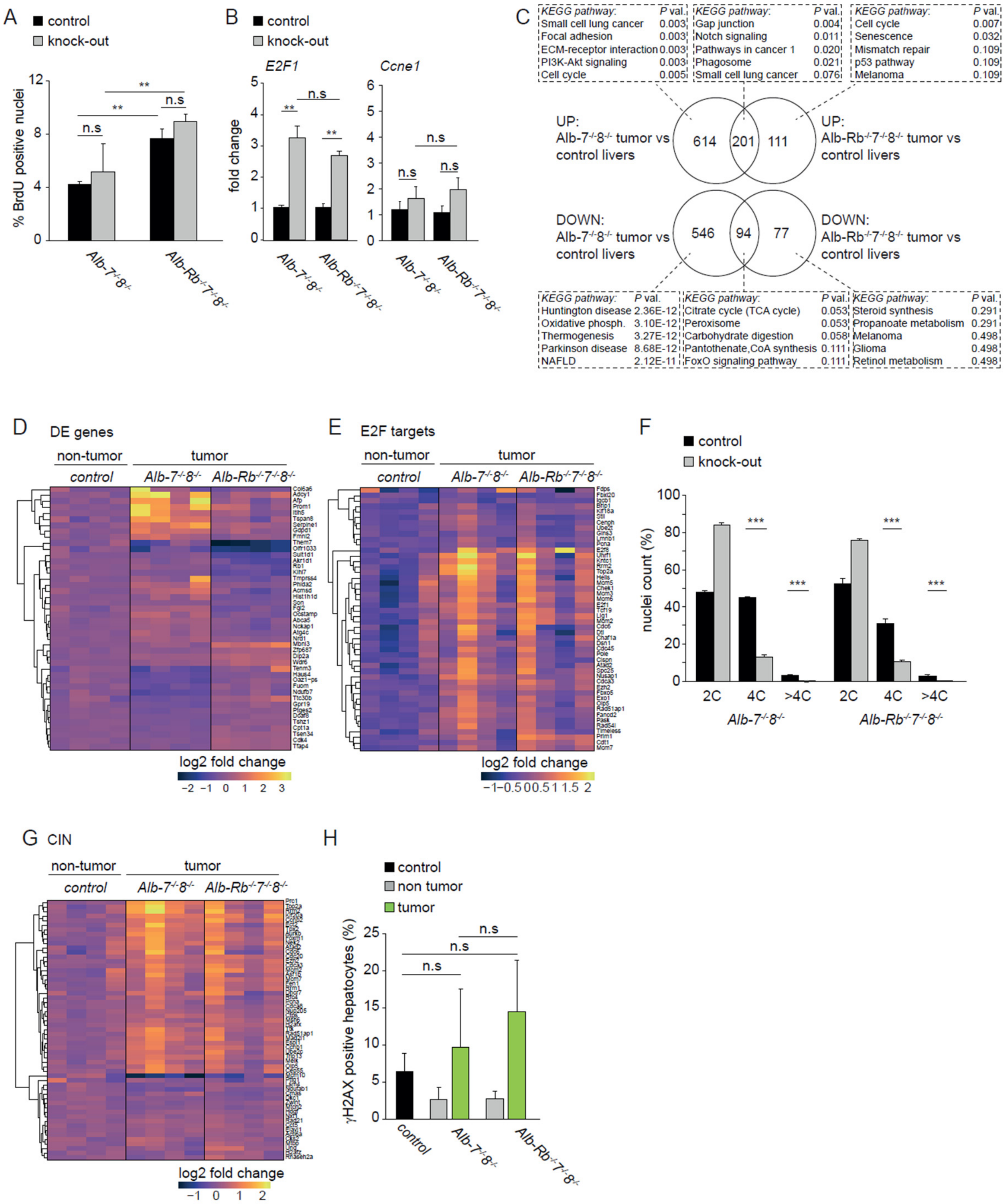

Figure S2: Loss of RB results in deregulation of cell cycle control in atypical E2F-deficient liver tumors. (**A**) Quantification of the percentage of BrdU-positive nuclei in control (Cre-negative) and knockout (Cre-positive) livers from *Alb-7-/-8-/-* and *Alb-Rb-/-7-/-8-/-* mice, 4 weeks after birth, measured by FACS. Data represent average ± SEM (*n*= 7-14 mice). (**B**) Transcript levels of E2F targets from *Alb- 7-/-8-/-* and *Alb-Rb-/-7-/-8-/-* 4 weeks old mice. Fold changes were adjusted to average of controls and Actb and Gapdh were used to normalize the expression. Data represent average ± SEM (*n*= 4-5 mice). (**C**) Venn diagram showing KEGG pathway enrichment analysis from the genes significantly up- or down-regulated in liver tumor samples from *Alb- 7-/-8-/-* and *Alb-Rb-/-7-/-8-/-* mice compared to non-tumor control livers in RNA sequencing (n=4 mice/ condition). *P val.*= Benjamini-Hochberg-corrected P value. (**D**) RNA-sequencing analysis of transcripts that were significantly differentially expressed in *Alb-Rb-/-7-/-8-/-* versus *Alb- 7-/-8-/-* tumor samples. Color scale represents log2-fold changes relative to the normal control livers in RNA sequencing (*n*=4). (**E**) RNA-sequencing data showing expression of E2F targets in samples from the indicated genotypes (*n*=4 livers/genotype). Color scale represents log2-fold changes relative to age-matched wild-type livers (*n*=4). (**F**) Quantification of 2C, 4C and >4C nuclei in 4 weeks old livers

from control and knock-out *Alb-7-/-8-/-* and *Alb-Rb-/-7-/-8-/-* mice. Data represent average ± SEM (*n*= 7-14 mice). (**G**) RNA sequencing data showing expression of CIN (chromosomal instability)-associated genes (signature CIN70) from the indicated genotypes (*n*=4 livers/ genotype). Color scale represents log2-fold changes relative to age-matched wild-type livers (*n*=4). (**H**) Quantification of ϒ-H2AX immunohistochemistry in 5 fields (40x objective) in tumor and adjacent non tumor areas of the livers from the indicated genotypes. Controls represent Alb-Cre negative littermates analyzed at the same time point as Alb-Cre positive ones. The data are presented as average ±SEM (1 tumor per mice was analyzed/mouse; *n*= 6 mice).

Data information: in (A, B, F and H) n.s not significant, *p<0.05, **p<0.01,***p<0.001 (Mann-Whitney Rank Sum test).

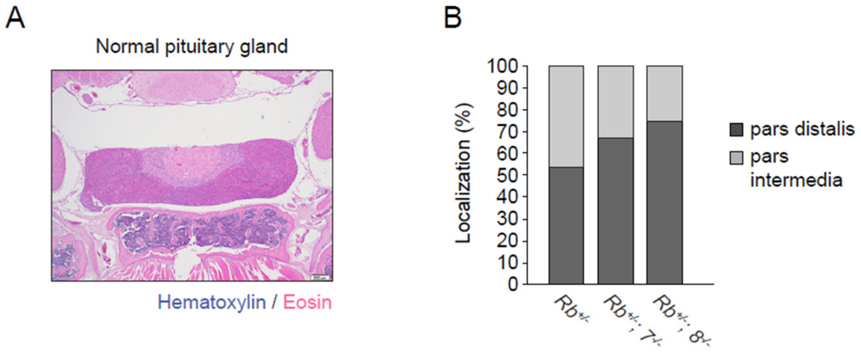

C

Incidence of hyperplasia								
Genotype	Thyroid gland *n*	%	Adrenal gland *n*	%		Mammary gland *n*	%	
Rb+/-	6/42	14	10/41	24	n.s	14/41	34	n.s
Rb+/-; *7*-/-	0/15	0	2/14	14		1/14	7	
Rb+/-; *8*-/-	0/20	0	5/19	26		8/21	38	
7-/-	0/9	0	1/9	11		2/9	22	
8-/-	0/11	0	3/11	27		3/11	27	

Figure S3: Effect of E2F7 and −8 deletion in pituitary glands of RB heterozygous mice. (**A**) Representative HE-stained image of a normal mouse pituitary gland. (**B**) Percentage of the location of the pituitary tumors in the indicated genotypes. (**C**) Incidence of hyperplasia in the additional neuroendocrine glands analyzed by a board-certified veterinary pathologist. n.s non significant (Chi-square)

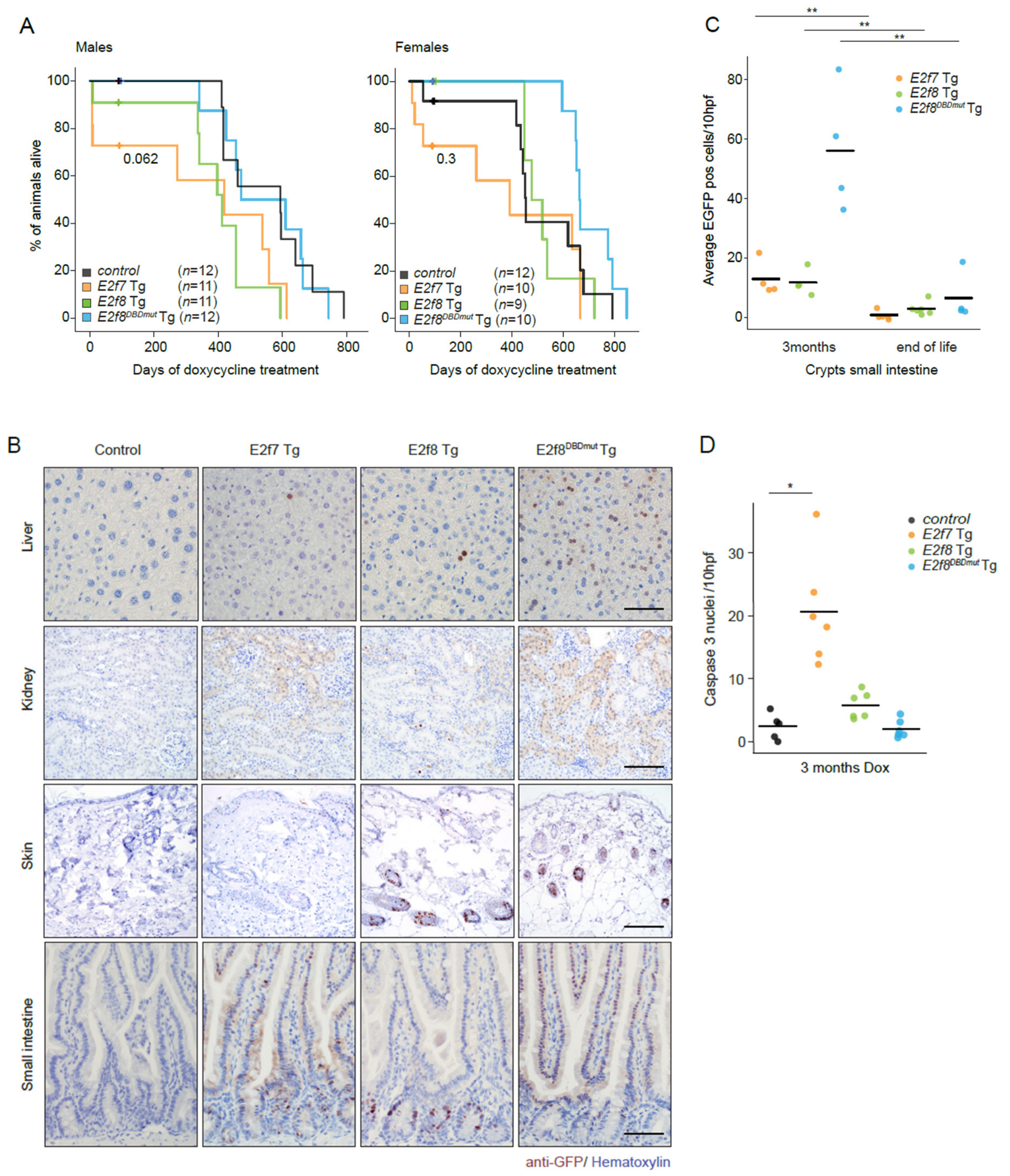

Figure S4: Overexpression of atypical E2Fs affects rapidly proliferating tissues in mice. (**A**) Kaplan-Meier overall survival of transgenic mice, split by gender. (**B**) Representative anti-EGFP immunohistochemistry pictures in the indicated tissues showing transgene expression in the indicated genotypes after 3 months of doxycycline treatment. Scale bars: 100µm. (**C**) Dot plot representing the counts of EGFP-positive cells in ten fields (40x objective) of the small intestinal crypts of 3 months-old and mice harvested at the end of life. Cross black line represents average. **p<0.01 (Mann-Whitney Rank Sum Test). (**D**) Dot plot representing the counts of Caspase 3-positive nuclei in ten fields (40x objective) of the small intestinal crypts of mice treated for 3 months with doxycycline. Cross black line represents average. *p<0.05 (Kruskal Wallis One Way Analysis of Variance on Ranks and Dunnett's Method for Multiple Comparisons vs. control).

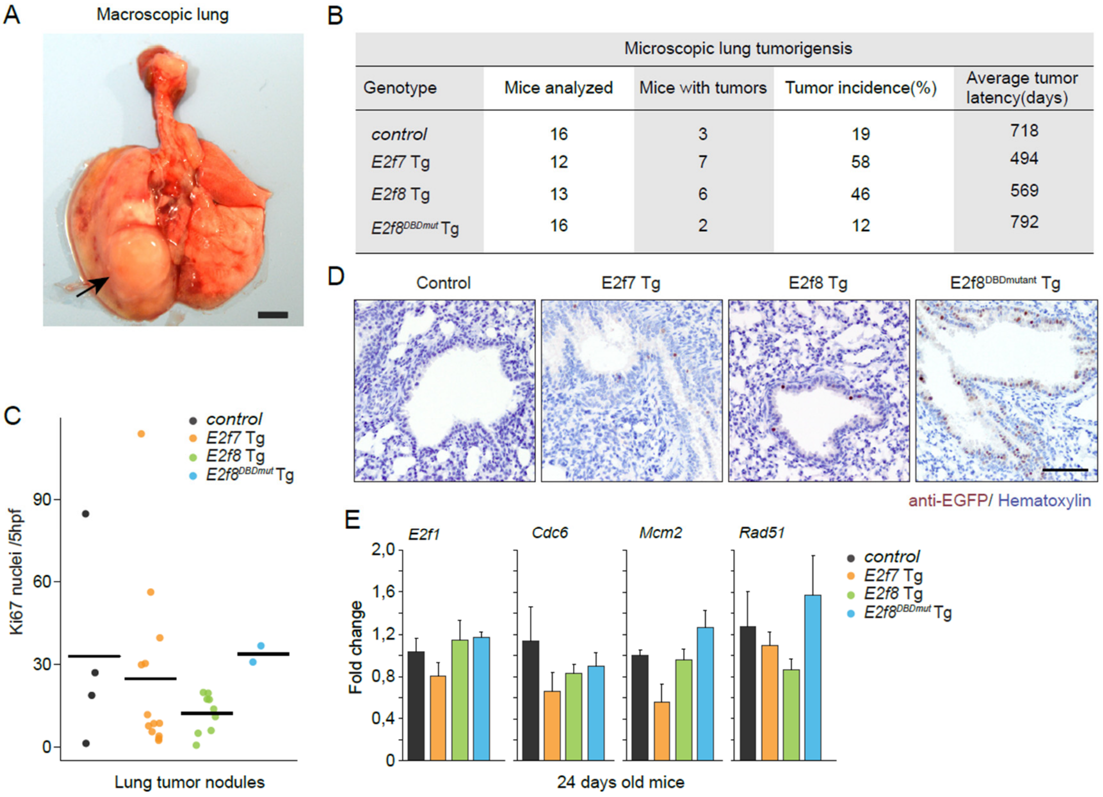

Microscopic lung tumorigensis				
Genotype	Mice analyzed	Mice with tumors	Tumor incidence(%)	Average tumor latency(days)
control	16	3	19	718
E2f7 Tg	12	7	58	494
E2f8 Tg	13	6	46	569
E2f8^{DBDmut} Tg	16	2	12	792

Figure S5: E2F7 or −8 overexpression promotes spontaneous lung tumorigenesis. (**A**) Representative image of a macroscopic lung lesion. Arrow indicates a lung lesion. Scale bar: 50mm. (**B**) Microscopic lung tumor incidence (%) and latency (days) of the indicated genotypes. Tumor incidence was determined on mice at the end of their life span. (**C**) Dot plot representing the counts of Ki67-positive nuclei in five fields (40x objective) of lung tumor nodules from the different genotypes. Cross black line represents average/ tumor nodule (*control* (*n*=4); *E2f7* Tg (*n*=13); *E2f8* Tg (*n*=9); *E2f8^{DBDmut}*. Tg (*n*= 2) mice. (**D**) Representative anti-EGFP immunohistochemistry pictures to detect transgene expression in lungs of 24 days old mice treated 3d with doxycycline. Scale bar: 50µm. (**E**) Transcript levels of E2F targets in lungs of juvenile mice from the indicated genotypes treated for 3 days with doxycycline. Fold changes were adjusted to average of *controls* and *Gapdh* was used to normalize the expression. Data represent average ± SEM (*n*= 5 mice).

Table S1: The table list the 44 differentially expressed genes that were significantly up- or down- regulated in Alb-Rb-/-7-/-8-/- versus Alb- 7-/-8-/- tumors.

**Gene symbol**	**Gene name**	**Log2 fold change**
Upregulated		
Mbnl3	Muscle blind-Like Splicing Regulator 3	2,37
Tenm3	Teneurin Transmembrane Protein 3	1,83
Ttc30b	Tetratricopeptide Repeat Domain 30B	1,60
Ndufb7	NADH: Ubiquinone Oxidoreductase Subunit B7	1,10
Gpr19	G protein-coupled receptor 19	1,08
Tfap4	Transcription factor AP-4	1,03
Oaz1-ps	Ornithine decarboxylase antizyme 1, pseudogene	0,93
Fuom	Fucose mutarotase	0,90
Haus4	HAUS augmin-like complex, subunit 4	0,75
Tsen34	tRNA splicing endonuclease subunit 34	0,74
Cdk4	Cyclin-dependent kinase 4	0,72
Wdr6	WD repeat domain 6	0,71
Ptges2	Prostaglandin E synthase 2	0,65
Dip2a	Disco interacting protein 2 homolog A	0,64
Cpt1a	Carnitine palmitoyltransferase 1A	0,63
Zfp687	Zinc finger protein 687	0,60
Tshz1	Teashirt zinc finger family member 1	0,55
Dcaf8	DDB1 and CUL4 associated factor 8	0,45
Downregulated		
Son	Son DNA binding protein	-0,38
Nrd1	Nardilysin, N-arginine dibasic convertase, NRD convertase 1	-0,48
Nckap1	NCK-associated protein 1	-0,75
Abca5	ATP-binding cassette, subfamily A (ABC1) member 5	-0,76
Klhl7	Kelch-like 7	-0,85
Atg4c	Autophagy related 4C, cysteine peptidase	-0,86
Rb1	RB transcriptional corepressor 1	-1,02
Akr1d1	Aldo-keto reductase family 1 member D1	-1,04
Sult1d1	Sulfotransferase family 1D member 1	-1,09
Fmnl2	Formin-like 2	-1,14
Gdpd1	Glycerophosphodiester phosphodiesterase domain containing 1	-1,31
Fgl2	Fibrinogen-like protein 2	-1,40
Acmsd	Amino carboxymuconate semialdehyde decarboxylase	-1,64
Serpine1	Serine (or cysteine) peptidase inhibitor, clade E, member 1	-1,80
Tspan8	Tetraspanin 8	-2,39
Olfr1033	Olfactory receptor 1033	-2,53
Ocstamp	Osteoclast stimulatory transmembrane protein	-2,66
Itih5	Inter-alpha (globulin) inhibitor H5	-2,70

Prom1	Prominin 1	-2,77
Phlda2	Pleckstrin homology like domain, family A, member 2	-2,86
Them7	Thioesterase superfamily member 7	-2,94
Adcy1	adenylate cyclase 1	-3,20
Hist1h1d	H1.3 linker histone, cluster member	-3,24
Tmprss4	Transmembrane protease, serine 4	-3,29
Afp	Alpha fetoprotein	-3,43
Col6a6	Collagen, type VI, alpha 6	-3,72

Table S2: mouse qPCR primers

	Forward primer (5′-3′)	***Reverse primer (3′-5′)***
Actb	AGCTCCTTCGTTGCCGGTCCA	TTTGCACATGCCGGAGCCGTTG
Cdc6	AGTTCTGTGCCCGCAAAGTG	AGCAGCAAAGAGCAAACCAGG
Cdt1	ACAGCCGGGCAAGATCCCCT	GGCTCCCAACTTCCGTGCCC
Ccnb1	AAAGGGAAGCAAAAACGCTAGG	TGTTCAAGTTCAGGTTCAGGCTC
Ccne1	AGCGAGGATAGCAGTCAGCC	GGTGGTCTGATTTTCCGAGG
Ccna2	CTTCTTCCTTTTCCCTTGGC	TTTCAGAGTCCCAGTGACCC
E2f1	ACATCACCAATGTCCTGGAGGG	AGCCGCTTACCAATCCCCAC
E2f7	GATGCGTTCGTGAACTCCCTG	AGAAACTTCTGGCACAGCAGCC
E2f8	GAGAAATCCCAGCCGAGTC	CATAAATCCGCCGACGTT
Gapdh	GAAGGTCGGTGTGAACGG	TGAAGGGGTCGTTGATGG
Mcm2	TCTCTCTCAGCATCTAGCCCTG	AGACTCATCTTCAAATGGGGG
Rad51	CTCATGCGTCAACCACCAG	GCTTCAGGAAGACAGGGAGAG
Rb1	TCTCCAGGGTAACCATACTGC	CAAGGGAGGTAGATTTCAATGG
